# Dynamic 3D echocardiography in virtual reality

**DOI:** 10.1186/1476-7120-3-37

**Published:** 2005-12-23

**Authors:** Annemien E van den Bosch, Anton HJ Koning, Folkert J Meijboom, Jackie S McGhie, Maarten L Simoons, Peter J van der Spek, Ad JJC Bogers

**Affiliations:** 1 Department of Cardiology, Erasmus MC University Hospital, Dr. Molewaterplein 40, 3015 GD Rotterdam, The Netherlands; 2 Bioinformatics, Erasmus MC University, Dr. Molewaterplein 50, 3015 GE Rotterdam, The Netherlands; 3 Cardiothoracic surgery, Erasmus MC University Hospital, Dr. Molewaterplein 40, 3015 GD Rotterdam, The Netherlands

## Abstract

**Background:**

This pilot study was performed to evaluate whether virtual reality is applicable for three-dimensional echocardiography and if three-dimensional echocardiographic 'holograms' have the potential to become a clinically useful tool.

**Methods:**

Three-dimensional echocardiographic data sets from 2 normal subjects and from 4 patients with a mitral valve pathological condition were included in the study. The three-dimensional data sets were acquired with the Philips Sonos 7500 echo-system and transferred to the BARCO (Barco N.V., Kortrijk, Belgium) I-space. Ten independent observers assessed the 6 three-dimensional data sets with and without mitral valve pathology. After 10 minutes' instruction in the I-Space, all of the observers could use the virtual pointer that is necessary to create cut planes in the hologram.

**Results:**

The 10 independent observers correctly assessed the normal and pathological mitral valve in the holograms (analysis time approximately 10 minutes).

**Conclusion:**

this report shows that dynamic holographic imaging of three-dimensional echocardiographic data is feasible. However, the applicability and use-fullness of this technology in clinical practice is still limited.

## Introduction

Evaluation of intracardiac anatomy from multiple two-dimensional echocardiographic images requires a mental conceptualisation process that is complicated by cardiac dynamics [1-3]. Currently, real-time 3D echocardiographic images of the heart do no longer demand this difficult and individually variable conceptualisation processes, by offering an equivocal presentation of cardiac anatomy throughout the cardiac cycle. However, the full 3D potential of these imaging modalities cannot be appreciated, since the 3D data are presented on a flat 2D screen. Virtual dynamic systems, known as virtual reality, can assist with the interpretation of 3D data of the heart in space and makes it possible to 'dive' into the 3D model of the heart [4-8]. This study is an attempt in the technological process of the future to evaluate whether virtual reality is feasible for 3D echocardiography and if 3D echocardiographic images in a virtual reality can advance to a clinically useful tool.

## Methods

Data sets from normal subjects and from patients with mitral valve disease, referred for a diagnostic echocardiogram, were selected with the aim of gathering a representative series of mitral valve pathological conditions with sufficient image quality. For this feasibility study, we selected 3D data sets of clinical conditions which have advantage of 3D perspective: (1) two patients with a normal mitral valve, (2) a patient with a mitral valve prolaps of the P2 segment of the posterior leaflet, (3) a patient with mitral valve stenosis, (4) a patient with hypertrophic obstructive cardiomyopathy and systolic anterior motion of the mitral valve, and (5) a patient with an atrioventricular septal defect (AVSD) where there is a commissure present between the superior and inferior bridging leaflets. Ten observers (5 cardiologist, 3 cardiologist-in-training and 2 cardiothoracic surgeons), who were blinded for the type of mitral valve morphology, were instructed to assess mitral valve anatomy/pathology and function.

### Three-dimensional echocardiographic data acquisition

The 3D data sets were acquired with the Philips Sonos 7500 echo system (Philips Medical Systems, Andover, MA, USA) equipped with a 3D data acquisition software package. Real-time 3D echocardiographic (RT-3DE) acquisition was done with ECG gating and in an end-expiratory breath-hold, lasting 6 to 8 seconds (depending on the heart rate). The 3D image data was stored on CD-ROM in DICOM 3.0 format and transferred to the computer (SGI Onyx4 Ultimate Vision, Silicon Graphics, Inc., Mountain View, CA, USA) driving the I-space.

### Visualisation in a virtual reality environment

**Figure 1:**
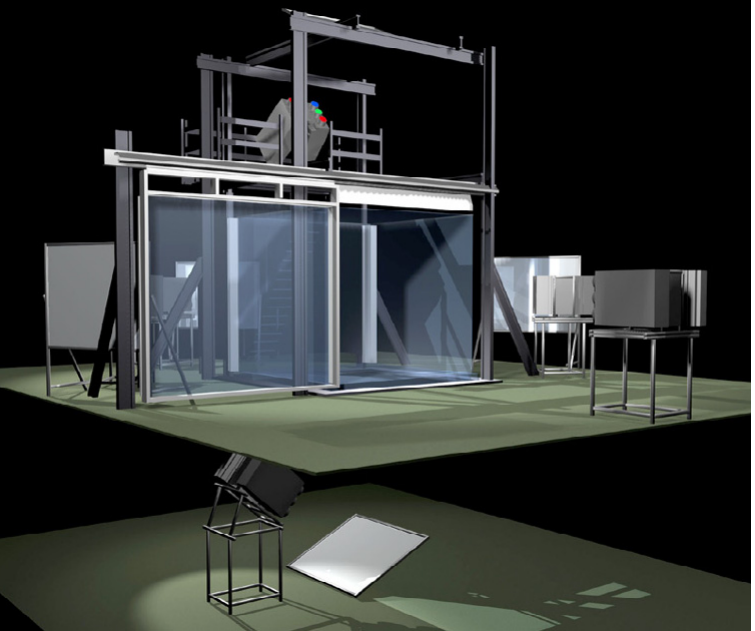
An impression of a 6-walled I-space virtual reality system (image courtesy of Barco N.V.). The I-space installed at the Erasmus is a 4-walled system, without ceiling and sliding back wall.

The BARCO (Barco N.V., Kortrijk, Belgium) I-space installed at the ErasmusMC, is a so-called four-walled CAVE(tm)-like virtual reality system (figure 1). In the I-space researchers are surrounded by computer-generated stereo images, which are projected by 4 high quality DLP-projectors on three walls and the floor of a small "room". The virtual reality system has a resolution of 1280 by 1024 pixels per projector. This is comparable to or greater than the resolution of the CRT monitors and LCD flat panels used in ultrasound systems and with workstations. The CAVORE (CAve VOlume REnderer) volume rendering application is used to investigate 3D ultrasound images during the cardiac cycle [9]. In the I-space, this result in an animated "hologram" of the dataset being visualised, floating in space in front of the viewers. The viewers wear a pair of lightweight glasses with polarising lenses that allows seeing the hologram with depth. Interaction with this "hologram" is by means of a virtual pointer and makes it possible to assess the interior of the heart (additional file 1).

The observers were instructed to create several cut plane in the hologram. For the analysis of the mitral valve, two opposite views were reconstructed: 1) a view from the left atrium towards the atrioventricular junction, allowing a "surgical view" of the mitral valve, and 2) a view from the left ventricular apex toward the mitral valve. The observers were asked to assess in these views, the anterior and posterior leaflets, and subvalvular apparatus for possible pathology (additional file 2).

## Results

**Figure 2:**
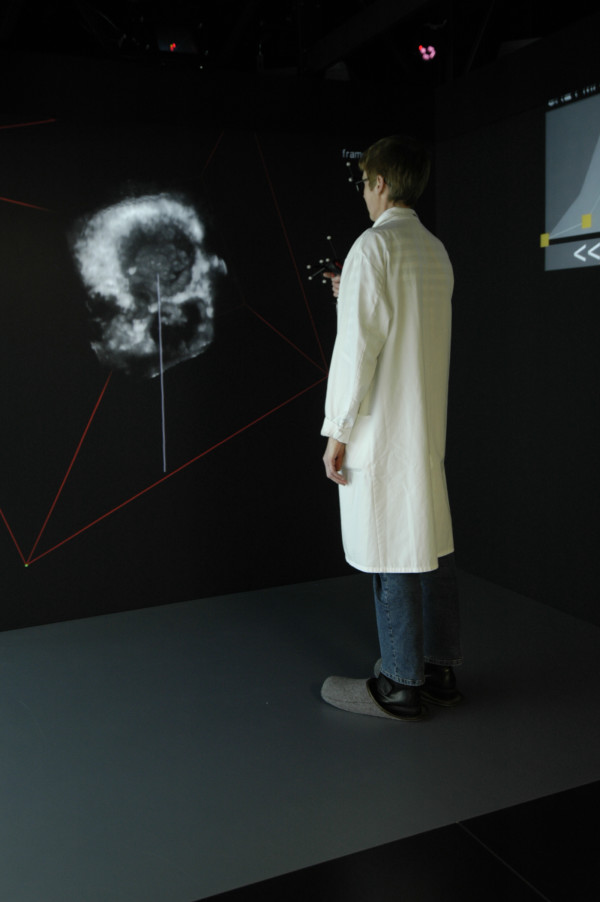
This figure shows a researcher in the I-Space, looking at the 3D hologram wearing a lightweight pair of glasses with polarising lenses. Within the I-space the head and hand movements of the viewer is being tracking by four infrared cameras, allowing a natural interaction with the images that are displayed.

The 3D data sets of the 6 patients with normal or abnormal mitral valve anatomy were transformed to 3D holographic heart models. Once the transformation method had been developed, the time required to create a hologram from the 3D data set varied from 1 to 3 minutes. Figure 2 demonstrates the reviewer inside the I-space creating the necessary cut planes in a hologram. After 10 minutes instruction in the I-Space, all observers could use the virtual pointer and make the necessary cut planes. The 10 independent observers correctly assessed the normal and pathological mitral valve in holograms with analysis time of approximately 10 minutes for each study.

### Visualisation of the mitral valve in virtual reality

**Figure 3:**
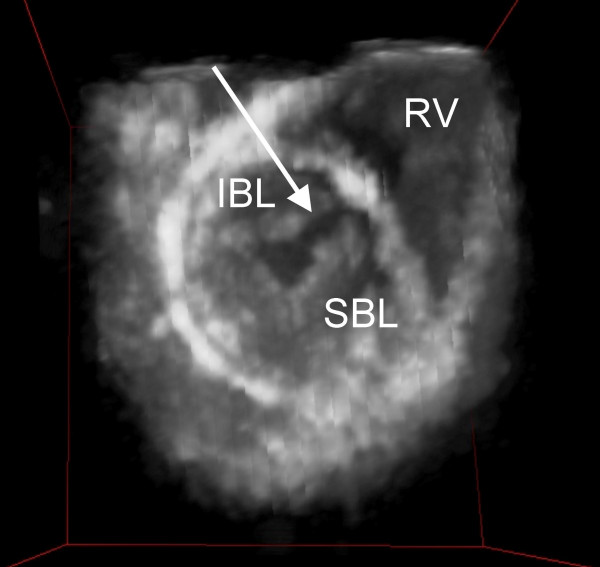
A 3D hologram of a patient with an atrioventricular septal defect is seen from a ventricular view. The arrow points out the commissure between the superior (SBL) and inferior bridging leaflets (IBL) (RV = right ventricle).

In the generated 3D hologram of a normal mitral valve (additional file 3), the mitral valve is best visualised from the apex of the left ventricle (LV) looking upwards to the crux of the heart. The anatomy of the mitral valve can clearly be discerned. Mitral valve motion becomes more apparent when the hologram is tilted up or down and can be stopped in any desired phase of the cardiac cycle. In the patient with localised prolaps of the posterior leaflet, this prolaps was best seen when looking down from the left atrium towards the mitral valve. In early systole, the mitral valve closes and the localised prolaps of the posterior mitral valve leaflet starts to become visible. With the time progression during systole, the extent of the prolaps is seen to increase to its maximum late in systole. The additional structures of the mitral valve apparatus (chordae, papillary muscles and valve leaflets) were visualised and identified by all observers. In figure 3, the left-sided atrioventricular (AV) valve of a heart with an atrioventricular septal defect is displayed, visualised from the apex of the LV. All observers correctly identified the commissure between the superior and inferior bridging leaflets.

## Discussion

This report presents a novel approach for visualisation of dynamic 3D echocardiographic data, known as virtual reality. The 3D echocardiographic data sets generated by a commercial available echo machine can be visualised as a dynamic hologram inside the I-Space. Until now, the 3D echocardiographic reconstructions could only be seen on a 2D screen, but virtual reality makes it possible to 'dive' into the actual 3D anatomy of the heart. We show that professionals, familiar with intracardiac anatomy, can learn how to handle the technique and cut through these holograms within 10 minutes. Subsequently, they were all able to correctly diagnose the intracardiac anatomy or pathology of the mitral valve. At the moment, I-Space technology is only available in a few dedicated research centres throughout the world. Therefore, the combination of the 3D echocardiography and virtual reality is very uncommon and the applicability and usefulness in clinical practice is still limited. However, in our opinion, it has potential and one can think of possible applications in the future.

Virtual reality provides a unique resource for education of intracardiac anatomy in general and/or specific cardiac structures. Especially for all professionals for whom detailed knowledge of the intracardiac anatomy is essential, virtual reality might lead to a better understanding of the intracardiac anatomy. With the growth of minimal invasive cardiac surgery and interventional procedures, the interest for simulation of the heart hologram as a training tool has increased. We believe that dynamic 3D echocardiography in virtual reality has the potential for wider applicability in providing a preview of real intracardiac anatomy. With the I-Space technology, the complex anatomy, pathology and dynamic changes of the heart are appropriately visualised in a virtual heart model, which increases the accessibility and availability of virtual reality for clinical practice. In order to be integrated into clinical practice, this application should be able to run on smaller virtual reality systems, either based on a single projection surface, or on a monitor (CRT or LCD).

## Competing interests

The author(s) declare that they have no competing interest.

## Authors' contributions

AEB conceived the study, and participated in acquisition and analysis of the data and drafted the manuscript. AK participated in acquisition and analysis of the data and drafted the manuscript. FM drafted the manuscript and revised it with important intellectual content. JM carried out the 3D echocardiographic assessments and participated in the drafting of the manuscript. MS revised the manuscript with important intellectual content. PS revised the manuscript with important intellectual content. AJB conceived the study, and revised the manuscript with important intellectual content and gave final approval of the version to be published. All authors read and approved the final manuscript.

## Supplementary Material

Additional File 1 Demonstration of the I-Space. The film shows a researcher going into the I-Space wearing a lightweight pair of polarised glasses and demonstrates the head/hand movements that allow a natural interaction with the images that are displayed. Click here for file

Additional File 2 Demonstration of a heart hologram in the I-Space. Click here for file

Additional File 3 Demonstration of a 3D echocardiogram from a normal mitral valve viewed from the left ventricular apex to the base of the heart. Click here for file
